# MLVA as a Tool for Public Health Surveillance of Human *Salmonella* Typhimurium: Prospective Study in Belgium and Evaluation of MLVA Loci Stability

**DOI:** 10.1371/journal.pone.0084055

**Published:** 2013-12-31

**Authors:** Véronique Wuyts, Wesley Mattheus, Guillaume De Laminne de Bex, Christa Wildemauwe, Nancy H. C. Roosens, Kathleen Marchal, Sigrid C. J. De Keersmaecker, Sophie Bertrand

**Affiliations:** 1 Department of Microbial and Molecular Systems, KU Leuven, Leuven, Belgium; 2 Platform Biotechnology and Molecular Biology, Scientific Institute of Public Health (WIVISP), Brussels, Belgium; 3 National Reference Centre for *Salmonella* and *Shigella*, Bacterial Diseases Division, Communicable and Infectious Diseases, Scientific Institute of Public Health (WIV-ISP), Brussels, Belgium; 4 Department of Plant Biotechnology and Bioinformatics, Ghent University, Gent, Belgium; 5 Department of Information Technology, Ghent University, IMinds, Gent, Belgium; Institut National de la Recherche Agronomique, France

## Abstract

Surveillance of *Salmonella enterica* subsp. *enterica* serovar Typhimurium (*S*. Typhimurium) is generally considered to benefit from molecular techniques like multiple-locus variable-number of tandem repeats analysis (MLVA), which allow earlier detection and confinement of outbreaks. Here, a surveillance study, including phage typing, antimicrobial susceptibility testing and the in Europe most commonly used 5-loci MLVA on 1,420 *S*. Typhimurium isolates collected between 2010 and 2012 in Belgium, was used to evaluate the added value of MLVA for public health surveillance. Phage types DT193, DT195, DT120, DT104, DT12 and U302 dominate the Belgian *S*. Typhimurium population. A combined resistance to ampicillin, streptomycin, sulphonamides and tetracycline (ASSuT) with or without additional resistances was observed for 42.5% of the isolates. 414 different MLVA profiles were detected, of which 14 frequent profiles included 44.4% of the *S*. Typhimurium population. During a serial passage experiment on selected isolates to investigate the *in vitro* stability of the 5 MLVA loci, variations over time were observed for loci STTR6, STTR10, STTR5 and STTR9. This study demonstrates that MLVA improves public health surveillance of *S*. Typhimurium. However, the 5-loci MLVA should be complemented with other subtyping methods for investigation of possible outbreaks with frequent MLVA profiles. Also, variability in these MLVA loci should be taken into account when investigating extended outbreaks and studying dynamics over longer periods.

## Introduction

*Salmonella* is the most frequent cause of food-borne outbreaks and human salmonellosis is the second most frequently reported zoonosis in the European Union [1]. The most common serovars of *Salmonella* isolated from human outbreaks are *Salmonella enterica* subsp. *enterica* serovar Enteritidis (*S.* Enteritidis) and *Salmonella enterica* subsp. *enterica* serovar Typhimurium (*S*. Typhimurium) [1]. Typing methods which allow characterization below the serovar level are essential in a surveillance program for this diverse genus. Classical surveillance programs for *Salmonella* rely on phenotyping methods such as phage typing and antimicrobial susceptibility testing. Nowadays, phage typing as a subtyping technique is often complemented with molecular methods like pulsed-field gel electrophoresis (PFGE), which is considered the gold standard for subtyping of *Salmonella* [2]. Yet, recent studies suggest that multiple-locus variable-number of tandem repeats analysis (MLVA) improves surveillance, detection of outbreaks and of sources of outbreaks of *Salmonella* and in particular of *S*. Typhimurium [3]–[6]. MLVA targets rapidly evolving genomic elements known as tandem repeats. This allows to use them to study genetic relatedness between isolates. There exist different MLVA schemes for *S.* Typhimurium, all with a different number of variable number of tandem repeat (VNTR) loci used. The first MLVA scheme for *S*. Typhimurium used 8 VNTR loci [7]. Improvements of PCR multiplexing and capillary electrophoresis to this MLVA scheme resulted in a scheme with 5 VNTR loci of which 3 loci were previously included in the 8-loci MLVA scheme and 2 loci were newly added [8], and which was equally well performing as the more labour intensive 8-loci MLVA scheme. This 5-loci scheme is currently in Europe the most commonly used MLVA scheme for *S*. Typhimurium [5], [9]. Variations on this 5-loci MLVA scheme have been tested. However, none of these showed to have large added value, compared to the 5-loci MLVA scheme, for routine surveillance and outbreak investigation of *S*. Typhimurium. Indeed, other MLVA schemes with 10 and 6 VNTR loci were developed for simultaneously tying of multiple *Salmonella* serovars [10], [11], thereby circumventing the disadvantage that most MLVA schemes are dedicated to one specific serovar. However, the small set of studied *S*. Typhimurium isolates only showed variability at the VNTR loci in common with the previously published 8-loci and 5-loci MLVA schemes. Additionally, a recent study which enlarged the 5-loci MLVA scheme with 11 additional VNTR loci for *S*. Typhimurium concluded that the 5-loci MLVA scheme was suitable to supplement PFGE in routine surveillance and outbreak investigation [12]. The 5-loci MLVA scheme of Lindstedt *et al*. [8] was recently validated in a large European inter-laboratory trial [5], [9]. Outside Europe other MLVA schemes are in use, *e.g.* PulseNet USA [13] developed a 7-loci MLVA protocol for *S*. Typhimurium by adding 2 VNTR loci to the 5-loci MLVA scheme. Whatever MLVA scheme used, the combination of the number of tandem repeats at a predefined number of MLVA loci results in a MLVA profile. Nonetheless, questions are raised related to the stability of MLVA loci [14], [15] and different ways of handling closely related MLVA profiles during outbreak investigations have been proposed [16]–[20].

The objective of this study was to evaluate the added value of MLVA typing for surveillance and outbreak detection by comparing MLVA profiles of a large *S*. Typhimurium collection to results obtained through traditional phenotyping methods and by evaluating the *in vitro* stability of the loci of the used MLVA scheme in a serial passage experiment.

The *S*. Typhimurium isolates in this study were collected in Belgium, where *S*. Typhimurium is since 2006 the serovar most frequently isolated from humans with an average of 1,985 isolates (55.9% of all *Salmonella* isolates, average from 2006 up to 2012) reported per year. *S*. Enteritidis follows with an average of 774 isolates (21.8%) reported each year [21].

For routine surveillance of human *S*. Typhimurium infections, the Belgian National Reference Centre for *Salmonella* and *Shigella* (NRCSS) uses phage typing and antimicrobial susceptibility testing. In outbreak situations, these phenotyping techniques are complemented with PFGE. During the 3-year period 2010–2012, the *S*. Typhimurium isolates were also analysed with the 5-loci MLVA scheme commonly used in Europe [5], [9]. Hence, this large collection of data created the ideal opportunity to investigate the potential and added value of MLVA for surveillance and outbreak detection of an important food-borne pathogen. This study demonstrates that although the discriminatory power of MLVA allows for an improvement of public health surveillance, additional or alternative molecular subtyping methods should be used to detect an outbreak and to uniquely characterize an outbreak isolate. Moreover, as some of the MLVA loci showed to be unstable, the interpretation of these genetic markers for subtyping should be done with caution.

## Materials and Methods

### Bacterial isolates

In Belgium, peripheral clinical laboratories collect *Salmonella* isolates from human patients and send them voluntarily to the NRCSS for serotyping. In the 3-year period from 1 January 2010 to 31 December 2012, the Belgian NRCSS received a total of 10,055 human *Salmonella* samples. From the 5,698 isolates (56.7%) that were serotyped as Typhimurium, a random subset of 1,439 *S*. Typhimurium isolates were analysed by phage typing, antimicrobial susceptibility testing and MLVA. Exclusion of 24 isolates which gave inconsistent phage types in confirmatory tests, led to a total of 1,415 randomly sampled *S*. Typhimurium isolates in this study (n_2010_ = 481, n_2011_ = 449, n_2012_ = 485). This randomly sampled set included 11 isolates (*i.e.* 0.8%) that were serotyped as the monophasic variant (4,[5],12:i:-) of *S*. Typhimurium. Five isolates collected during an outbreak in a day nursery in 2011 were also included in this study, so that the total size of the studied population is 1,420 isolates. All typing data are available as (dataset S1).

### Serotyping and phage typing

Serotyping of *Salmonella* isolates was performed by slide agglutination with commercial antisera by the Kauffmann-White scheme [22]. Phage typing of *S*. Typhimurium was carried out according to the recommendations of the Health Protection Agency (Colindale, United Kingdom) [23]. A frequent phage type was defined as a phage type that was detected in at least 50 isolates during the 3-year period 2010–2012.

### Antimicrobial susceptibility testing

The susceptibility to 13 antibiotics was determined by the disk diffusion (Kirby-Bauer) method following recommendations of the European Committee on Antimicrobial Susceptibility Testing (EUCAST) and using Bio-Rad (Nazareth, Belgium) disks [24]. Inhibition zones were interpreted according to EUCAST criteria [25] for ampicillin (A), amoxicillin plus clavulanic acid (Amc), cefotaxime (Ctx), chloramphenicol (C), ciprofloxacin (Cip), gentamicin (G), trimethoprim (Tmp) and trimethoprim plus sulfamethoxazole (Sxt), and Clinical and Laboratory Standards Institute's (CLSI) criteria for kanamycin (K), nalidixic acid (Na), streptomycin (S), sulphonamides (Su) and tetracycline (T). Quality control was performed using the *Escherichia coli* ATCC 25922 strain. Multidrug resistance (MDR) was defined as resistance to 4 or more antimicrobials.

### Multiple-locus variable-number of tandem repeats analysis (MLVA)

MLVA was performed as described previously [8]. Liquid cultures were heated at 95°C for 10 minutes and used directly in the PCR reaction after a brief centrifugation at 18,188 g for 10 minutes, or a DNA lysate was prepared by heating a single colony in 300 µl sterile water at 100°C for 10 minutes and collecting the supernatant after centrifugation at 9,300 g for 10 minutes. PCR products were subjected to capillary electrophoresis on a ABI 3130xl Genetic Analyzer (Life Technologies^TM^), after which the size of the PCR products was determined with GeneMapper® software v.1.0 (Life Technologies^TM^). GeneScan^TM^ 600 LIZ® (Life Technologies^TM^) was used as size standard. The calibration strains were the same as described previously [26] with the addition of two strains, STm-SSI32 and STm-SSI33 [27]. Presence of loci which presented a relatively low peak area with respect to other loci in the sample, was confirmed through single target PCR and agarose gel electrophoresis. MLVA profiles are reported as a string of 5 numbers (STTR9-STTR5-STTR6-STTR10-STTR3) representing the number of repeats at the corresponding locus or NA in case a PCR product was not obtained for that locus [26]. A frequent MLVA profile was defined as a MLVA profile that was detected in at least 20 isolates during the 3-year period 2010–2012. A MLVA profile that was detected in less than 20 isolates during the 3-year period 2010–2012 defined a rare profile.

### Stability experiment

The *in vitro* stability of the 5 MLVA loci was evaluated in 20 *S*. Typhimurium isolates with a frequent MLVA profile and a frequent phage type, and in 11 *S*. Typhimurium isolates with a rare MLVA profile but with a frequent phage type. A single colony from a culture grown overnight on LB agar at 37°C was inoculated into 5 ml LB broth and incubated overnight at 37°C without shaking. Next, a series of 50 passages at a rate of two passages per day was performed by inoculating 20 µl of culture into 5 ml fresh LB broth and incubating at 37°C without shaking. Glycerol (25% v/v) stocks (−80°C) were made before each 5^th^ passage. MLVA was performed on heated liquid cultures after every fifth passage, as described above, leading to a total of 310 typing tests [28].

### Minimum spanning tree and diversity indices

A minimum spanning tree based on MLVA profiles of *S*. Typhimurium isolates was created in BioNumerics 6.5 (Applied Maths) using the categorical coefficient and no priority rules for the algorithm.

The discriminatory power of phage typing, antimicrobial resistance testing and MLVA was evaluated in the 1,415 randomly sampled *S*. Typhimurium isolates with Simpson's index of diversity (*D*) [29] and Shannon's indices of diversity (*H*') and equitability (*E*) [30]. Shannon's indices were calculated with the Biodiversity Calculator developed by J. Danoff-Burg and C. Xu [31].

## Results

### Phage types

**Figure 1:**
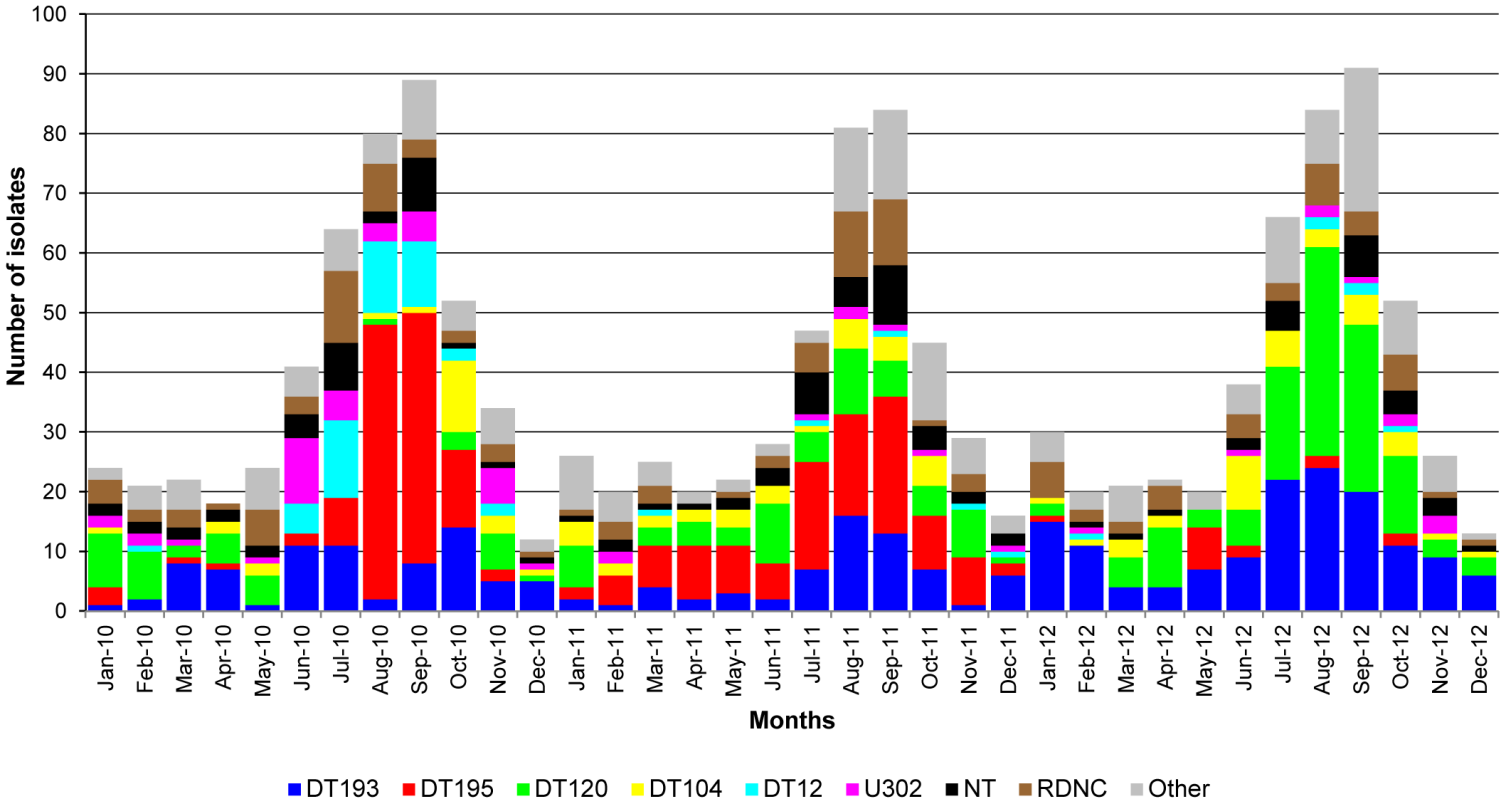
Monthly distribution of phage types among the *Salmonella* Typhimurium population over the 3-year period. N = 1,407 as for 13 isolates in this study the isolation date is unknown. NT: not-typable; RDNC: reacts-but-does–not-conform; Other: phage types with less than 3% occurrence in the sample population.

The 1,420 *S*. Typhimurium isolates were first subtyped by phage typing. Forty-one distinct phage types were present, nevertheless 969 (68.2% of the sample population) isolates were attributed to the frequent phage types DT193 (20.0%), DT195 (17.7%), DT120 (16.3%), DT104 (6.3%), DT12 (4.1%) and U302 (3.9%). The isolates with frequent phage types were not equally present over each year of the study period (Figure 1). Phage types DT12 and U302 were mainly found in 2010. Phage type DT195 dominated the seasonal peaks in 2010 and 2011, but was replaced by phage type DT120 during the seasonal peak in 2012. Nevertheless, isolates with phage types DT193 and DT104 were detected throughout the 3-year period.

Not-typable (NT) and reacts-but-does-not-conform (RDNC) isolates of *S*. Typhimurium comprised respectively 7.1% and 9.2% of the sample population. Other phage types covered each less than 3% of the sample population.

The monophasic variant isolates were phage typed as DT193 (6 isolates), RDNC (3), DT120 (1) and U302 (1).

### Antimicrobial susceptibility testing

Subsequently, the 1,420 *S*. Typhimurium isolates were further characterised by antimicrobial susceptibility testing. The isolates of *S*. Typhimurium were most frequently resistant to ampicillin (77.3% of the sample population), sulphonamides (63.0%), streptomycin (57.3%) and tetracycline (54.6%) whereas 11.9% of the isolates were susceptible to all antibiotics tested. Frequency of resistance to other antibiotics was between 0.2% for ciprofloxacin and 14.7% for trimethoprim. In our study population, the percentage of resistance remained stable over the 3-year period, except for the resistance to ampicillin and tetracycline, which showed a slightly increasing trend. An increasing trend was also observed for isolates with decreased susceptibility to sulphonamides. However, a declining trend was observed for decreased susceptibility to nalidixic acid (data not shown).

Multidrug resistance (MDR) occurred in 51.1% of the *S*. Typhimurium isolates. The dominant MDR pattern was ampicillin, streptomycin, sulphonamides and tetracycline (ASSuT) with or without additional resistances, which was responsible for 83.3% of the MDR isolates in the 3-year period and which was observed in 6 of the monophasic variant isolates (the 5 other monophasic variant isolates had a ASSu resistance pattern). ASSuT was also the most common resistance pattern in *S*. Typhimurium belonging to phage types DT193 and DT120. ACSSuT with or without additional resistances was the most frequent pattern for MDR isolates of phage types DT104 and DT12. A combined resistance to 6 or more antibiotics occurred regularly for phage types DT12, DT120 and DT104, which presented a moderate to high percentage of MDR isolates. On the other hand, *S*. Typhimurium with phage types U302 and DT195 presented a low frequency of MDR isolates and were mainly associated with a single resistance to ampicillin (Table 1).

**Table 1:** Overview of antimicrobial resistance and MLVA characteristics in relation to the phage type (n = 1,420).

Phage type	No. isolates (% of total)	No. susceptible isolates (%)	No. MDR isolates (%)	Most common resistance patterns (% of isolates with resistance pattern)	No. MLVA types	Most common MLVA profiles (STTR9-STTR5-STTR6-STTR10-STTR3) (% of isolates with MLVA profile)
DT193	284 (20.0)	4 (1.4)	156 (54.9)	ASSuT (35.6), A (28.9), ASSu (7.0)	79	3-12-10-NA-211 (7.7), 3-13-10-NA-211 (7.7)
DT195	251 (17.7)	11 (4.4)	82 (32.7)	A (47.8), ASSuT (14.3), ASSu (6.8)	72	3-12-10-NA-211 (10.0), 3-15-13-NA-311 (8.4)
DT120	231 (16.3)	11 (4.8)	187 (81.0)	ASSuT (48.1), ASSuSxtTTmp (15.6), ASSu (6.9)	76	3-12-9-NA-211 (11.7), 3-12-10-NA-211 (10.4)
DT104	90 (6.3)	2 (2.2)	75 (83.3)	AAmcCSSuT (33.3), ACSSuT (15.6), AAmcCNaSSuT (10.0)	70	3-12-21-14-NA (4.4), 3-15-10-23-410 (4.4)
DT12	58 (4.1)	8 (13.8)	30 (51.7)	ACSSuT (19.0), SSu (13.8), AAmcCSSuT (8.6), AAmcCNaSSuT (8.6)	50	3-13-13-16-311 (5.2), 3-14-19-16-NA (5.2)
U302	55 (3.9)	17 (30.9)	12 (21.8)	A (29.1), ASSu (5.5), T (5.5), ASuSxtTmp (5.5)	41	3-16-10-NA-311 (7.3), 4-9-12-9-211 (5.5)
NT	101 (7.1)	15 (14.9)	57 (56.4)	ASSuT (26.7), A (18.8), ASSuSxtTTmp (5.0)	60	3-13-10-NA-211 (12.9), 3-12-10-NA-211 (5.0), 3-15-11-NA-311 (5.0), 4-9-10-10-111 (5.0)
RDNC	131 (9.2)	37 (28.2)	33 (25.2)	ASSu (25.2), ASSuT (11.5), SSuT (6.9)	64	3-12-11-NA-211 (22.9), 3-17-9-NA-211 (4.6)
Other	219 (15.4)	64 (29.2)	93 (42.5)	ASSuT (25.6), T (9.1), A (6.8), ASSu (6.8)	117	3-12-10-NA-211 (7.8), 3-12-11-NA-211 (7.8)

MDR: multidrug resistant; MLVA: multiple-locus variable-number of tandem repeats analysis; NT: not-typable; RDNC: reacts-but-does-not-conform; Other: phage types with less than 3% occurrence in the sample population; A: ampicillin; Amc: amoxicillin plus clavulanic acid; C: chloramphenicol; Na: nalidixic acid; S: streptomycin; Su: sulphonamides; Sxt: trimethoprim plus sulfamethoxazole, T: tetracycline; Tmp: trimethoprim.

### MLVA typing

Among the 1,420 *S*. Typhimurium isolates typed with MLVA targeting 5 loci, 414 distinct MLVA profiles were detected. Absence of a PCR amplicon occurred most often at loci STTR10 (75.0% of the sample population), STTR3 (3.3%) and STTR6 (1.8%). The highest number of different alleles was seen at locus STTR6 (27), followed by loci STTR10 (24), STTR5 (22), STTR3 (20) and STTR9 (11). Thirty MLVA profiles were observed throughout the 3-year period, while 131, 93 and 108 MLVA profiles were only identified in 2010, 2011 and 2012, respectively.

263 rare MLVA profiles (63.5% of the MLVA profiles) were detected for only one *S*. Typhimurium isolate (18.5% of the sample population), while 14 frequent MLVA profiles (3.4% of the MLVA profiles) comprised 44.4% of the *S*. Typhimurium isolates.

**Figure 2:**
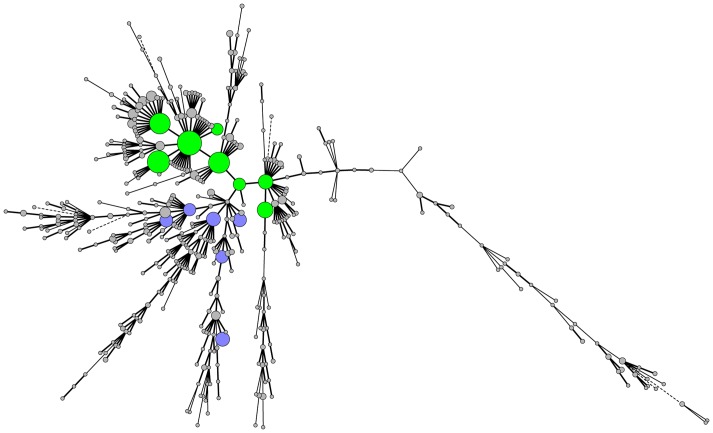
Minimum spanning tree calculated of MLVA profiles of *Salmonella* Typhimurium isolates over the 3-year period. N = 1,420. Each node represents a different MLVA profile. Node colour denotes frequent MLVA profiles of group 1 (green) and group 2 (blue), and rare MLVA profiles (grey). Node size is proportional to the number of isolates with that MLVA profile. Branch thickness indicates how many loci are different in the MLVA profiles of the connected nodes. Thick solid lines connect nodes that differ by 1 MLVA locus, thin solid lines connect nodes that differ by 2 MLVA loci and dashed lines connect nodes that differ by 3 MLVA loci. MLVA: multiple-locus variable-number of tandem repeats analysis.

**Figure 3:**
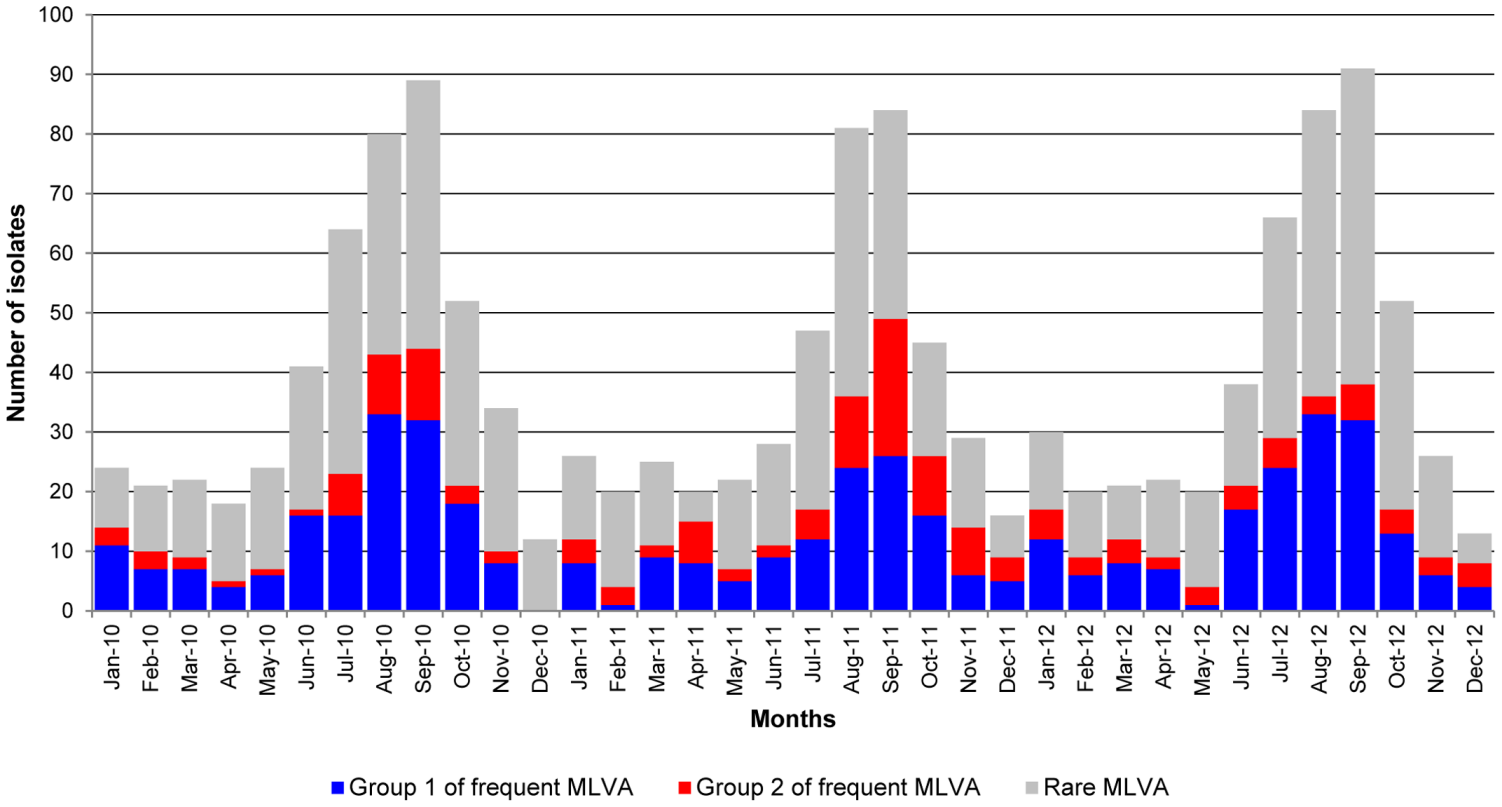
Monthly distribution of *Salmonella* Typhimurium isolates over the 3-year period according to MLVA profile. N = 1,407 as for 13 isolates in this study the isolation date is unknown. Group 1 of frequent MLVA profiles: 3-12-8-NA-211, 3-12-9-NA-211, 3-12-10-NA-211, 3-12-11-NA-211, 3-13-8-NA-211, 3-13-9-NA-211, 3-13-10-NA-211 and 3-13-11-NA-211. Group 2 of frequent MLVA profiles: 3-14-11-NA-311, 3-15-10-NA-311, 3-15-11-NA-311, 3-15-12-NA-311, 3-15-13-NA-311 and 3-16-10-NA-311. Rare MLVA: MLVA profiles that have less than 1.4% occurrence in the sample population. MLVA: multiple-locus variable-number of tandem repeats analysis.

In order to simplify the data analysis, we have decided to partition the frequent MLVA profiles into 2 groups taking into account the number of 27-bp repeats in locus STTR3: group 1 (32.0% of the sample population) with allele 211 and group 2 (12.3%) with allele 311 (Figure 2). The MLVA profiles of each group were single-locus and single-repeat variants of other MLVA profiles in the group. The frequent MLVA profiles were observed throughout the 3-year period (Figure 3), except for profile 3-15-13-NA-311, belonging to group 2, which was not detected in 2010. Nine monophasic variant isolates were typed with a MLVA profile belonging to group 1. Rare MLVA profiles 3-11-11-NA-211 and 3-11-3-NA-211 were detected in the other monophasic variant isolates.

**Figure 4:**
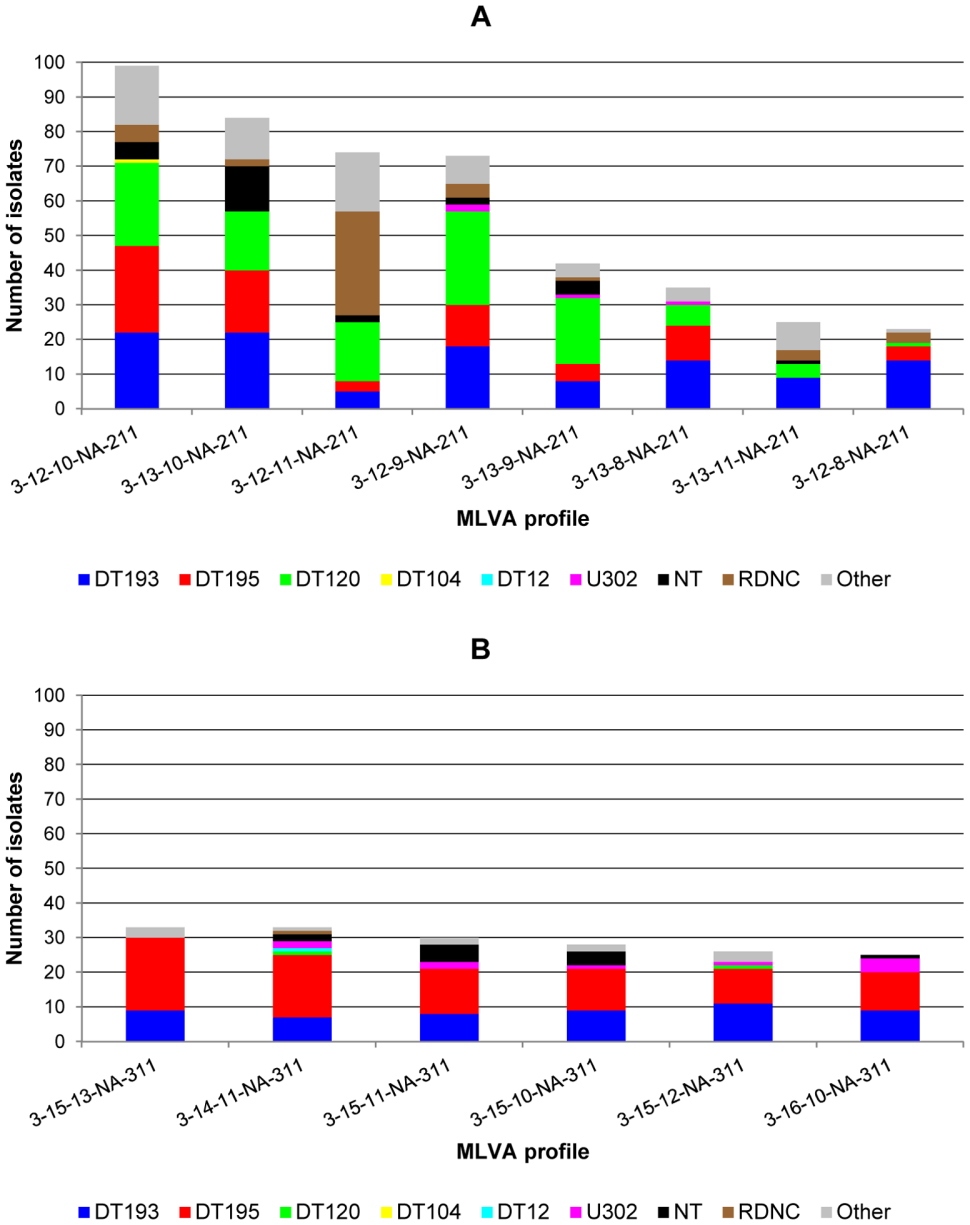
Prevalence of *Salmonella* Typhimurium isolates in this study with a frequent MLVA profile. Figure 4A: Group 1 of frequent MLVA profiles (n = 455) for which other phage types are DT7, DT20, DT56, DT110, DT116, DT138, DT185, DT194 and U311. Figure 4B: Group 2 of frequent MLVA profiles (n = 175) for which other phage types are DT35, DT41, DT99, DT194, DT208 and U311. MLVA: multiple-locus variable-number of tandem repeats analysis; NT: not-typable; RDNC: reacts-but-does–not-conform.

The *S*. Typhimurium isolates of both groups differed with respect to the presence of phage types (Figure 4). DT193, DT195, U302 and NT isolates were observed in both groups, whilst DT120, DT110, DT138, U311 and RDNC isolates were common in group 1 and scarce in group 2. Interestingly, 30 out of the 48 (62.5%) RDNC isolates in group 1 had MLVA profile 3-12-11-NA-211 (Figure 4). Frequent phage types DT104 and DT12 were observed only for a single isolate of group 1 and of group 2, respectively. Within the frequent phage types, the number of distinct MLVA profiles ranged from 41 for U302 up to 79 for DT193 (Table 1). Except for phage types DT104 and DT12, which showed many different MLVA profiles, the most common MLVA profile for each phage type belonged to the frequent MLVA groups.

Also with respect to antimicrobial resistance there were dissimilarities between both groups of frequent MLVA profiles. Whereas group 1 comprised 73.6% MDR isolates, group 2 included 6.9% MDR isolates. Correspondingly, the most common antimicrobial resistance patterns were ASSuT (50.4%) in group 1 and a single resistance to ampicillin (86.3%) in group 2. Both groups displayed equivalent numbers of isolates which were susceptible to all antibiotics tested (3.3% and 2.3% for group 1 and 2, respectively).

### Diversity of phage typing, antimicrobial susceptibility testing and MLVA

To compare the discriminatory power of MLVA to that of the phenotyping methods used, Simpson's and Shannon's diversity indices were calculated. The discriminatory power of a subtyping method is defined as the ability to distinguish between unrelated isolates [29] and the higher the value of a diversity index, the higher the discriminatory power of the subtyping method. Simpson's *D* ranges from 0 to 1 and gives the probability that 2 randomly sampled and unrelated isolates will have a different subtype [29]. Rare subtypes, which apply to only a small number of isolates, will have a small contribution to Simpson's index and as such, the number of subtypes has little influence on Simpson's index [32]. Shannon's *H*' is an indicator for subtype richness [32] and its highest value is *ln S*, where *S* is the number of subtypes. Shannon's *E* is a measure for the evenness of the subtype distribution [32] and has 1 as maximum value.

Calculated values for Simpson's *D*, Shannon's *H*' and *E* indices were, respectively, 0.88, 2.49 and 0.67 for phage typing, 0.88, 2.84 and 0.62 for antimicrobial susceptibility testing, and 0.98, 4.92 and 0.82 for MLVA. These values suggest that MLVA has a higher discriminatory power than both phage typing and antimicrobial susceptibility testing.

### Typing of outbreak isolates

To evaluate the added value of MLVA in case of outbreak detection, five isolates originating from an outbreak in a day nursery in 2011 were included in this study. These isolates were characterised as phage type DT138 with antimicrobial resistance pattern ASSu and MLVA profile 3-13-11-NA-211. Whereas each of these typing results independently are shared with isolates which are not related to the outbreak and hence would not allow to identify specific clusters of outbreak isolates, the combination of their MLVA profile and phage type is unique to the outbreak isolates compared to all other isolates in the 3-year period.

The outbreak isolates were also typed with PFGE (data not shown), which resulted in identical patterns for these isolates. However, this PFGE pattern could not be compared to other isolates in this study, as it was not feasible for the Belgian NRCSS to perform PFGE on all randomly sampled isolates during the 3-year period.

### Stability of MLVA loci

To evaluate the *in vitro* stability of the number of tandem repeats in the MLVA loci, 31 *S*. Typhimurium isolates were subjected to a serial passage experiment. The isolates selected for this stability experiment covered all frequent phage types and 17 different MLVA profiles, of which 6 and 3 MLVA profiles belonged to respectively frequent MLVA groups 1 and 2 (Table 2).

**Table 2:** Overview of isolates and outcome of the stability experiment (n = 31).

ID number	Phage type	Initial MLVA profile (STTR9-STTR5-STTR6-STTR10-STTR3)	Frequent MLVA group	Varying number of repeats observed (passage of first occurrence)
11–1129	DT104	3-12-9-NA-211	1	-
12–2475	DT193	3-12-9-NA-211	1	-
12–3110	DT193	3-12-10-NA-211	1	STTR6: 11 (25)
11–0841	DT120	3-12-11-NA-211	1	-
11–2577	DT195	3-12-11-NA-211	1	-
12–3096	DT193	3-13-8-NA-211	1	-
11–1058	DT193	3-13-9-NA-211	1	-
12–0828	DT120	3-13-9-NA-211	1	-
12–1779	DT120	3-13-9-NA-211	1	-
11–0050	DT120	3-13-10-NA-211	1	-
11–2038	DT120	3-13-10-NA-211	1	-
11–2650	DT195	3-13-10-NA-211	1	-
11–2847	DT195	3-13-10-NA-211	1	-
11–3000	U302	3-15-11-NA-311	2	-
12–1651	DT195	3-15-11-NA-311	2	-
11–2321	DT195	3-15-13-NA-311	2	STTR6: 12 (45)
11–3418	DT193	3-15-13-NA-311	2	STTR6: 12 (35)
11–3445	DT193	3-15-13-NA-311	2	-
11–2326	U302	3-16-10-NA-311	2	-
12–1918	U302	3-16-10-NA-311	2	-
11–2630	DT12	3-14-10-NA-311	-	STTR5: 13 (10)
11–0676	DT195	3-14-10-NA-311	-	-
11–3355	DT104	3-14-11-21-311	-	STTR10: 20 (35)
11–1160	DT104	3-14-18-14-311	-	-
10–02975	DT12	3-14-18-14-311	-	STTR10: 9 (15); STTR10: 7 (50); STTR10: 17 (50)
11-0008	DT104	3-15-10-23-311	-	-
11–0210	DT104	3-15-10-23-311	-	STTR6: 11 (30)
11–0444	U302	3-18-16-17-311	-	STTR10: 20 (20)
11–0335	U302	4-14-18-7-211	-	-
11–3448	DT12	5-13-15-8-211	-	-
11–3005	DT12	5-14-11-8-211	-	STTR9: 3 (10); STTR5: 13 (5); STTR6: 10 (10); STTR6: 12 (20)

MLVA: multiple-locus variable-number of tandem repeats analysis.

Among the 20 isolates with a frequent MLVA profile, 3 isolates (15.0%) presented a single-repeat variant at locus STTR6 during the serial passage experiment. Among the *S*. Typhimurium with a rare MLVA profile, variations of the initial MLVA profile were observed in 6 out of 11 isolates (54.5%). These variations were not only noticed in locus STTR6, but also in loci STTR5, STTR9 and STTR10. In one isolate with a rare MLVA profile, variations of the initial MLVA profile were observed in loci STTR5, STTR6 and STTR9, and another isolate with a rare MLVA profile presented 3 different variations at locus STTR10 (Table 2). For loci STTR6 and STTR5 only single-repeat variants were seen, whereas the varying allele for locus STTR9 differed 2 repeats from the original allele and for locus STTR10 there were differences from 1 up to 7 repeats between original and varying alleles.

## Discussion

*S*. Typhimurium is the most frequently isolated serovar from human patients in Belgium and hence subtyping of this serovar is very important for outbreak detection and tracing outbreak sources. The Belgian NRCSS relies on phage typing and antimicrobial susceptibility testing for routine surveillance of *S*. Typhimurium, complemented with PFGE during outbreak investigations. PFGE, which is widely considered as the gold standard for subtyping of *Salmonella*, is a labour intensive and time consuming technique and therefore implementation of this subtyping method for routine surveillance is not realisable for the Belgian NRCSS. MLVA, which requires less hands-on time and allows faster typing and easy inter-laboratory comparison of results, has been adopted by several European countries for surveillance and detection and investigation of outbreaks [5]. For evaluation of the capability of MLVA typing for surveillance and outbreak detection of human *S*. Typhimurium in Belgium, 1,420 isolates collected over the 3-year period 2010–2012 were characterised by phage typing, antimicrobial susceptibility testing and 5-loci MLVA.

Our study shows that phage types DT193, DT195, DT120, DT104, DT12 and U302 dominated the *S*. Typhimurium population. This predominance of a small number of phage types was also observed in other countries [3], [4], [33], [34] and reduces the capacity of phage typing to discriminate outbreak isolates. Additionally, 16.3% of the isolates are categorized as RDNC or NT, which lowers the proportion of isolates that are subtyped by this technique, and hence its suitability for surveillance and outbreak detection.

Antimicrobial susceptibility testing is another phenotyping method used in public health surveillance. In this study, ASSuT is the leading antimicrobial resistance pattern, which is observed with or without additional resistances for 42.5% of the *S*. Typhimurium isolates. The ASSuT pattern has been reported in France, UK, Spain, Luxembourg, Italy and Germany in association with DT193, DT120 and NT isolates and is often connected to the monophasic variant of *S*. Typhimurium [33], [35]–[40]; but also, differently from Belgium, in combination with phage type U302 in Denmark and Italy [41], [42]. Contrary to other European countries [1], [37], [39], [43], Belgium has a low rate (0.8% of the randomly sampled set) of human monophasic variant isolates of *S*. Typhimurium.

Molecular techniques like MLVA are generally considered to improve surveillance and detection of outbreaks and their sources because of different advantages of this method. The possibility to present the result as a string of numbers is one of the strengths of MLVA and allows easy sharing across country borders and setup of databases of MLVA profiles [5], [26]. Yet, laboratories have to agree on the set of calibration strains and on the nomenclature used, so that different laboratories report the same MLVA profiles for the same isolates [9], [44]. Another already reported asset of MLVA subtyping is the high discriminatory power of this technology [5], [6]. MLVA targeting 5 loci divided the investigated *S*. Typhimurium collection into 414 distinct profiles and indeed allowed for discrimination within isolates of the same phage type, which is in concordance with previous studies [3], [8], [15], [34]. However, 14 frequent MLVA profiles included 44.4% of the *S*. Typhimurium isolates. These 14 profiles were partitioned into 2 groups based on the number of 27-bp repeats in locus STTR3. The two MLVA groups differ regarding associated phage types and frequency of MDR isolates. Most of the MLVA profiles in the first group have already been described in other European countries [18], [34], [40], [43], [45] and are present in the MLVA-NET database [46]. Of the second group, 2 profiles have been reported in literature [34], [47] and 1 additional profile has been found in the MLVA-NET database [46].

As in our study almost half of the *S*. Typhimurium population is characterised by only 14 different MLVA profiles, while the total population is represented by 414 distinct MLVA profiles, questions on the discriminating ability of this subtyping technique could be raised. In order to compare the discriminatory power in an objective manner, diversity indices were calculated for the different subtyping methods used in this study. A higher Simpson's index of diversity (*D*) was obtained for MLVA as compared to phage typing and antimicrobial susceptibility testing, but the latter techniques showed the same *D* value. This demonstrates the small influence of the number of subtypes on Simpson's diversity index, as antimicrobial susceptibility testing revealed 99 distinct patterns compared to only 41 detected phage types, while resulting in equal discriminatory power according to Simpson's index. Shannon's diversity (*H*') and equitability (*E*) indices, which are indicators of the number of subtypes and of the evenness of the distribution of these subtypes [32], respectively, offer a more differentiated measure for comparison of discriminatory power, *i.e.* aiming at a high number of evenly distributed subtypes. Shannon's *H*' denotes that antimicrobial susceptibility testing was more able to discriminate between unrelated isolates than phage typing, but Shannon's *E* values indicate that phage types are more equally distributed than antimicrobial resistance patterns. Both Simpson's and Shannon's indices suggest that MLVA has a higher discriminatory power than phage typing and antimicrobial resistance testing, as was concluded from earlier studies [3], [8], [32], [34], and which would result in an improved surveillance and detection of possible outbreaks. However, as *S*. Typhimurium isolates in this study are dominated by 14 frequent MLVA profiles and 6 frequent phage types, care must be taken with the interpretation of these indices. *S*. Typhimurium isolates with a frequent MLVA profile show from 4 up to 11 different phage types and visa-versa, *S*. Typhimurium isolates with a frequent phage type show from 41 up to 79 different MLVA profiles. This would implicate that all isolates with a same frequent MLVA profile or all isolates with a same frequent phage type do not originate from a single *S*. Typhimurium strain. Consequently, for detection of an outbreak or the source of an outbreak with isolates with a frequent MLVA profile or phage type, when using the in this study applied 5-loci MLVA, a combination of both subtyping techniques might be necessary, which was also previously observed [15], [18]. This was also the case for the 5 outbreak isolates presented in this study. On the contrary, MLVA or phage typing might be sufficient to distinguish a cluster of isolates with rare MLVA profiles or phage types. However, for locating the source of a human outbreak in animal, food or environmental isolates, we must take into account that rare MLVA profiles or phage types in human isolates might be common MLVA profiles or phage types in animals, food or the environment [3], which would complicate the designation of a single source.

In addition to the discriminatory power of a subtyping method, also the stability of the assessed markers should be taken into account. From our serial passage experiment on 31 *S*. Typhimurium isolates, we observed that 71.0% of the MLVA profiles remain stable *in vitro*. However, variations due to passages occurred and most variations were seen among isolates with a rare MLVA profile, which presented variations in more isolates and at more loci. Single-locus variants constituted 8 of the 9 isolates with varying alleles. MLVA loci STTR 6 and STTR5 displayed only single-repeat variants, in contrast to STTR9 and STTR10, where differences up to 7 repeats from the original number of repeats were detected. Locus STTR3 remained stable throughout the experiment, which was also observed in a serial passage experiment by Barua *et al.* [48] on 4 DT41 *S*. Typhimurium isolates of poultry origin. In contrast to the results of our experiment, in which more sampling points were included, the MLVA profiles of their DT41 isolates remained stable at locus STTR9 and only single-repeat variants at locus STTR10 and differences up to 4 repeats at locus STTR6 were detected [48]. Nevertheless, these passage experiments point out that we cannot rule out the possibility that isolates with closely related MLVA profiles are not clonal.

In conclusion, based on Simpson's and Shannon's indices, 5-loci MLVA has a higher discriminatory power for the 1,420 *S*. Typhimurium collected during the 3-year period 2010–2012, and can thus improve public health surveillance. However, outbreak detection with MLVA is not straightforward, since for isolates with a frequent MLVA profile, phage typing is still necessary to achieve a unique, combined subtyping result in this study. Also, during investigation of extended outbreaks, variations in MLVA profiles should be taken into account, since *in vitro* stability could not be confirmed for all 5 MLVA loci. Therefore, improvement of the 5-loci MLVA scheme may be desirable. Indeed, the MLVA scheme which is currently implemented in Europe, and which has been used in this study, targets 5 VNTR loci of which 1 locus did not amplify in 75.0% of the 1,420 isolates and 4 loci showed instabilities during a serial passage experiment. Since there are more than 30 VNTR loci described in literature for *S*. Typhimurium [7], [8], [10]–[13], [49], there may be possibilities to improve the assay. Other opportunities may be the further development and implementation of CRISPR genotyping [47] for subtyping of *S*. Typhimurium, so that the need of a second subtyping assay for genetically homogeneous populations is eliminated, or the development of a subtyping assay that combines different typing methods in a single molecular assay with stable markers.

## Supporting Information

Dataset S1 **Typing data of the studied** ***Salmonella*** **Typhimurium population.** n = 1,420. A: ampicillin; Amc: amoxicillin plus clavulanic acid; C: chloramphenicol; Cip: ciprofloxacin; Ctx: cefotaxime; G: gentamicin; I: intermediate; K: kanamycin; MLVA: multiple-locus variable-number of tandem repeats analysis; Na: nalidixic acid; NT: not-typable; R: resistant; RDNC: reacts-but-does-not-conform; S: sensitive/streptomycin; Su: sulphonamides; Sxt: trimethoprim plus sulfamethoxazole; T: tetracycline; Tmp: trimethoprim; \N: unknown isolation date/absence of a PCR amplicon in MLVA.
(CSV)
